# Association of Baseline Comorbidities With First-Year Adherence to GLP-1 Receptor Agonists in Patients With Diabetes or Obesity: A Retrospective Cohort Study

**DOI:** 10.1177/10600280251384637

**Published:** 2025-12-07

**Authors:** Ziyang Mai, John Kornak, Suzanne M. Dufault, Michael W. Strand, Andrew R. Reikes, Jonathan H. Watanabe

**Affiliations:** 1 Department of Epidemiology and Biostatistics, University of California, San Francisco, CA, USA; 2 Department of Clinical Pharmacy, University of California, San Francisco, CA, USA; 3 Divisions of General Internal Medicine and Endocrinology, Department of Medicine, UC Irvine School of Medicine, Irvine, CA, USA

**Keywords:** glucagon-like peptide-1 receptor agonist, GLP-1 RA, adherence, comorbidity, type 2 diabetes, diabetes, obesity

## Abstract

**Background:**

Glucagon-like peptide-1 receptor agonists (GLP-1 RAs) are increasingly used for diabetes and obesity. Yet, the role of comorbidities on adherence, and variability due to drug subtype or indication remains understudied.

**Objective:**

Study objective was to (a) evaluate association between baseline comorbidities and digestive system adverse events on first-year GLP-1 RA adherence and (b) assess how associations differ across GLP-1 RA subtypes and between patients with diabetes and obesity.

**Method:**

We conducted a retrospective cohort study of adults with type 1 or type 2 or obesity initiating GLP-1 RAs between 2018 and 2023 using the University of California Health Data Warehouse. Primary outcome was first-year adherence association with baseline comorbidity and digestive system adverse event status. Adherence odds ratios based on exposure were estimated using regression. Analyses were stratified by indication and drug type.

**Result:**

Among 69 049 adults who initiated a GLP-1 RAs between 2018 and 2023, 59.3% had diabetes and 74.4% had obesity. Among patients with diabetes, hypertensive disorder (OR: 1.06, 95% CI: 1.01-1.10), hyperlipidemia (OR: 1.17, 95% CI: 1.12-1.22), and chronic kidney disease (CKD) (OR: 1.14, 95% CI: 1.08-1.21) increased adherence likelihood. Among patients with obesity, hyperlipidemia (OR: 1.10, 95% CI: 1.05-1.15) and CKD (OR: 1.09, 95% CI: 1.02-1.16) were associated with increased adherence. In patients with diabetes, atherosclerotic cardiovascular disease (ASCVD) and digestive system adverse events reduced adherence likelihood (OR: 0.90, 95% CI: 0.86-0.95) and (OR: 0.94, 95% CI: 0.90-0.98), respectively. Results were similar for patients with obesity. Findings remained consistent overall in brand-specific analyses.

**Conclusion and Relevance:**

Comorbidities affected GLP-1 RA adherence with variation by drug and indication. Further research is needed to understand drivers of these patterns and how they may inform future strategies to support adherence.

## Introduction

Glucagon-like peptide-1 receptor agonists (GLP-1 RAs) are an increasingly fundamental class of antihyperglycemic and weight management agents with expanding indications in both endocrinology and cardiometabolic care. Originally approved for glycemic control in patients with type 2 diabetes mellitus, GLP-1 RAs have gained attention for their ability to promote sustained weight loss and reduce cardiovascular risk.^1-3^ By mimicking endogenous GLP-1 activity, these agents enhance glucose-dependent insulin secretion, suppress inappropriate glucagon release, slow gastric emptying, and increase satiety. Notably, their mechanism of action leads to a lower risk of hypoglycemia compared with other glucose-lowering medications, making them particularly suitable for individuals at risk of hypoglycemia-related complications.^1,2^ The cardiovascular and renal benefits of GLP-1 RAs have also been well-documented in randomized clinical trials, prompting their inclusion in major guideline recommendations: Evidence from the LEADER trial showed that liraglutide reduced cardiovascular events—including cardiovascular death, non-fatal myocardial infarction, non-fatal stroke—as well as progression of diabetic nephropathy compared with placebo. Consistent findings across other landmark randomized trials of GLP-1 RAs (SUSTAIN-6, ELIXA, EXSCEL, Harmony Outcomes, REWIND, and PIONEER 6) further underscore the cardiometabolic benefits of this drug class.^4^ The American Diabetes Association now advises the use of GLP-1 RAs for patients with type 2 diabetes and a history of atherosclerotic cardiovascular disease (ASCVD), heart failure, or chronic kidney disease (CKD) to reduce the risk of future complications. Their low risk of hypoglycemia and positive effect on body weight further support their role in managing patients with high cardiometabolic burden.^1^

GLP-1 RA use is rapidly expanding, with a large nationwide study showing a nearly 9-fold increase in users from 2011-2014 to 2019-2023, and a large, diverse, state-wide dataset confirming exponential growth over the same period. In both studies, the rise was primarily observed in non-Hispanic, White females with obesity.^5,6^ Despite these benefits and the rapid growth in prescribing rates, real-world uptake and sustained use of GLP-1 RAs remain limited.^7-10^ High cost, gastrointestinal intolerance, and insurance barriers have been widely cited as contributors to underutilization.^7-9^,^11,12^ Equally concerning is the issue of medication adherence. Real-world data showed that only 35% to 45% of GLP-1 RAs users maintain adequate adherence over a 12-month period, commonly defined by a proportion of days covered (PDC) ≥0.8.^13,14^ Among patients using GLP-1 RAs for weight loss, adherence is even more variable, with discontinuation often occurring within the first 6 months of therapy.^14^ Poor adherence not only compromises the glycemic and weight-reduction benefits of GLP-1 RAs but may also reduce their potential cardioprotective and renoprotective effects.^10,15^ In an extensive observational study, non-adherent patients had a 58% higher risk of all-cause hospitalization (OR: 1.58, 95% CI: 1.38-1.81) and an 81% higher risk of all-cause mortality (OR: 1.81, 95% CI: 1.46-2.23) compared with adherent patients.^16^ Similarly, premature discontinuation of pharmacotherapy for obesity has been associated with weight regain and worsening metabolic parameters.^10^ Given the growing demand for GLP-1 RAs and the cost implications of these therapies, understanding and addressing adherence barriers is essential for improving clinical outcomes and ensuring sustainable health care delivery.

While multiple factors influencing GLP-1 RA adherence have been explored, including age, sex, socioeconomic status, injection burden, and dosing frequency, the potential role of patients’ baseline comorbidity profiles has received comparatively less attention. Existing studies often include comorbidities as covariates for statistical adjustment, but rarely treat them as exposures and predictors of interest for adherence outcomes.^8,9,12,17^ Yet, clinical experience suggests that certain comorbid conditions may influence patients’ motivation to remain adherent or may interact with tolerability, complexity of care, or perceived medication benefit. For example, a patient with coexisting CKD or cardiovascular disease may perceive a greater urgency to adhere to treatment and may also derive additional therapeutic benefit compared with low-risk patients. Clarifying these relationships could improve our ability to target adherence interventions, support clinical decision-making, and optimize long-term outcomes for diverse patient populations.

The objective of this research was to examine the association between specific pre-existing health conditions and 12-month adherence to GLP-1 RAs, and to assess whether these associations vary by clinical indication (type 2 diabetes or obesity) and medication subtype, with the goal of informing future adherence interventions and prescribing strategies.

## Methods

### Study Design and Data Source

We conducted a retrospective cohort study using data from the University of California Health Data Warehouse (UCHDW), a centralized, de-identified electronic health record repository maintained by UC Health. The UCHDW integrates longitudinal clinical data from 18 health professional schools, 6 medical centers, and 10 hospitals covering a large and demographically diverse patient population across California.^18,19^ The data are harmonized under the Observational Medical Outcomes Partnership (OMOP) Common Data Model, enabling standardized multi-site research.^20^ As of 2024, the database included records for over 10 million patients dating back to 2012.^18^

### Cohort Selection

We identified adults (≥18 years) who were new users of GLP-1 RAs between January 1, 2018, and December 31, 2023, for the GLP-1 RA brands Ozempic, Rybelsus, Victoza, Mounjaro, Trulicity, Wegovy, Saxenda, and Bydureon. Given that FDA-approved indications are brand specific irrespective of generic active ingredient, we performed analyses by brand and describe findings in terms of GLP-1 RA brand throughout this manuscript. New users were defined as individuals with no prior record of GLP-1 RA prescriptions before the window of study, and must have at least one health care encounter during that period to ensure baseline data in the 6 months prior to their first GLP-1 RA prescription. Hence, at a minimum, all study patients had not received a GLP-1 RA in at least 6 months prior to index use in the study. Eligible participants were required to have a diagnosis of type 1 or type 2 at any point before initiation to be considered as having diabetes, or a diagnosis of obesity in the 12 months prior to be considered to have obesity. Continuous follow-up was confirmed by requiring at least one health care encounter more than 12 months post-initiation. The final cohort was followed for a 12-month study period after the date of their first GLP-1 RA prescription.

### Outcome Measurement

The primary outcome was medication adherence in the 12-month period following index new of use of the GLP-1 RA, measured by the PDC. PDC was calculated as the total number of days supplied with any GLP-1 RA during the follow-up period, divided by 365 days. Patients were considered adherent if their PDC equaled or exceeded 0.80. For analyses stratified by brand, only the days supplied with the same medication initiated at baseline were included in the PDC calculation.

### Exposure

The primary exposures were the pre-existing comorbidities at baseline of hypertensive disorder, hyperlipidemia, heart failure, CKD, and ASCVD as well as baseline presence of digestive system adverse events. These were identified using diagnosis records prior to the earliest GLP-1 RA prescription indexed by ICD-10 codes (See Appendix B).

### Adjustment Variables

Regression covariates included age, sex, race, ethnicity, and clinical indication. In subgroup analyses, indication was defined as the presence of obesity in the diabetes cohort and the presence of diabetes in the obesity cohort.

### Statistical Analysis

Descriptive statistics summarized baseline characteristics for the full cohort and by clinical indication of diabetes and obesity. Categorical variables were compared using chi-square tests, and continuous variables using *t* tests.

Multiple logistic regression models were used to estimate adjusted odds ratios (ORs) and 95% confidence intervals (CIs) for the association between baseline comorbidities and adherence to GLP-1 RAs (defined as PDC ≥ 0.8). Models were adjusted for age, sex, race, ethnicity, and clinical indication. Analyses were stratified by clinical indication, defined as obesity status within the diabetes cohort and diabetes status within the obesity cohort. All comorbidities were entered into the models additively.

We conducted a secondary analysis stratified by GLP-1 RA brand to explore adherence differences across medications. This approach accounts for variations in FDA approval timing, dosing frequency, and approved indications, which may affect the measurement of outcome.

All analyses were conducted in R version 4.4.0 (R Core Team, Vienna, Austria) in the Databricks (Databricks, San Francisco, CA, USA) environment. SQL queries were executed in the Databricks environment to extract and filter data, which were processed in R using the SparkR and dplyr packages. Results were visualized using ggplot2 and formatted with the assistance of knitr and kableExtra. The research database used is a Health Insurance Portability and Accountability Act (HIPAA) Limited Data Set operationalized by UC Health as “non-human subjects research” and analyses are considered institutional review board exempt.^21^ Accordingly, Human Ethics and Consent to Participate declarations are not applicable. The Strengthening the Reporting of Observational Studies in Epidemiology (STROBE) guidelines were followed.^22^

## Results

**Figure 1.**
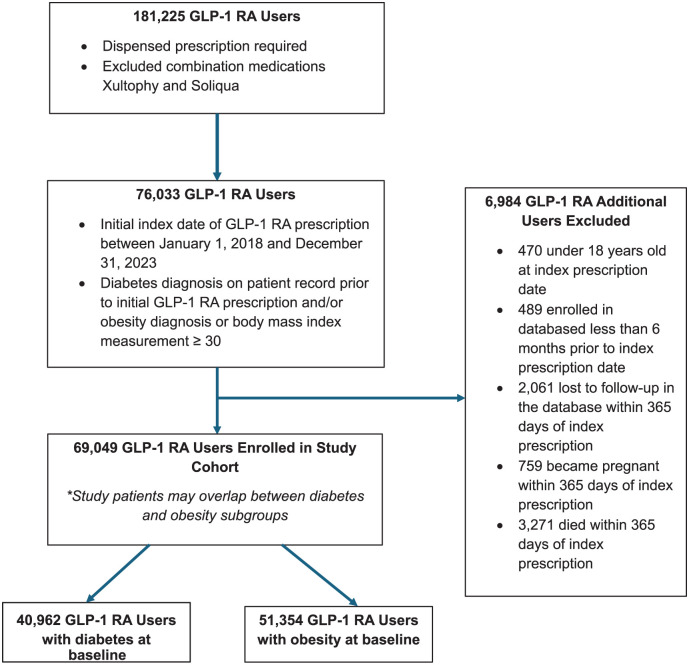
Cohort selection diagram.

Among 69 049 adults who initiated GLP-1 RAs with a diagnosis of diabetes and/or obesity, 59.3% had a diabetes diagnosis, and 74.4% had an obesity diagnosis. A total of 23 267 individuals (33.7%) had both conditions (see Figure 1). The mean age at the time of first GLP-1 RA prescription was 56.7 years (Standard deviation [SD]: 13.9), and 58.5% were female. Most users were White (51.9%), followed by Hispanic or Latino (25.1%), Asian (8.1%), and Black (6.8%).

Patients with diabetes were older (mean age, 59.9 years; SD, 13.0) and less frequently female (51.2%) compared with those with obesity (mean age, 54.8 years; SD, 13.9; 62.0% female). Comorbidities were more prevalent in the diabetes group, notably CKD (16.6% vs 3.7%).

During the first year of treatment, 40.5% of all users adhered to GLP-1 RAs, and 36.7% remained adherent to the same product. Adherence was higher in the diabetes group (45.0% overall; 41.5% same-drug adherence) than in the obesity group (39.4% overall; 35.6% same-drug adherence; see Table 1).

**Table 1.** Baseline Characteristics of GLP-1 Receptor Agonist Initiators, Group by Diabetes and Obesity.

	All GLP users with diabetes or obesity	GLP users with diabetes	GLP users with obesity
N	69 049 (100.00%)	40 962 (59.32%)	51 354 (74.37%)
Diabetes	40 962 (59.32%)	N/A	23 267 (56.80%)
Obesity	51 354 (74.37%)	23 267 (45.31%)	N/A
Age at first drug use (mean [SD])	56.65 [13.93]	59.87 [12.99]	54.77 [13.87]
**Gender**			
Female	40 404 (58.51%)	20 951 (51.15%)	31 851 (62.02%)
Male	28 604 (41.43%)	20 002 (48.83%)	19 465 (37.90%)
**Race**			
White	35 834 (51.90%)	19 907 (48.60%)	27 820 (54.17%)
Asian	5579 (8.08%)	4469 (10.91%)	2817 (5.49%)
Black	4703 (6.81%)	2901 (7.08%)	3753 (7.31%)
**Ethnicity**			
Hispanic or Latino	17 329 (25.10%)	11 376 (27.77%)	13 118 (25.54%)
**Adherence outcome**			
Adherent in the first year	27 938 (40.46%)	18 429 (44.99%)	20 253 (39.44%)
Adherent to the same brand in first year	25 371 (36.74%)	16 993 (41.48%)	18 259 (35.56%)
**Baseline comorbidity**			
Hypertensive disorder	33 294 (48.22%)	26 414 (64.48%)	22 485 (43.78%)
Hyperlipidemia	29 375 (42.54%)	23 639 (57.71%)	20 176 (39.29%)
ASCVD	13 145 (19.04%)	11 031 (26.93%)	8600 (16.75%)
Heart failure	4803 (6.96%)	4085 (9.97%)	3453 (6.72%)
Chronic kidney disease	7559 (10.95%)	6780 (16.55%)	1881 (3.66%)
Digestive system adverse events	22 737 (32.93%)	15 217 (37.15%)	17 554 (34.18%)
**Brand of GLP-1 RA used at initiation**			
Ozempic	25 077 (36.32%)	14 500 (21.00%)	19 640 (28.44%)
Trulicity	17 993 (26.06%)	14 937 (21.63%)	10 783 (15.62%)
Rybelsus	7097 (10.28%)	5283 (7.65%)	4582 (6.64%)
Wegovy	7411 (10.73%)	625 (0.91%)	7292 (10.56%)
Victoza	4416 (6.40%)	3141 (4.55%)	2951 (4.27%)
Saxenda	3289 (4.76%)	338 (0.49%)	3236 (4.69%)
Mounjaro	2183 (3.16%)	808 (1.17%)	1916 (2.77%)
Bydureon	1447 (2.10%)	1222 (1.77%)	861 (1.25%)

### Association of Baseline Comorbidities With Adherence

Among users with diabetes, hypertensive disorder (OR: 1.06, 95% CI: 1.01-1.10), hyperlipidemia (OR: 1.17, 95% CI: 1.12-1.22), and CKD (OR: 1.14, 95% CI: 1.08-1.21) were each associated with increased odds of adherence. In contrast, ASCVD (OR: 0.90, 95% CI: 0.86-0.95) and digestive system adverse events (OR: 0.94, 95% CI: 0.90-0.98) were associated with decreased adherence. Heart failure association was non-statistically significant with estimated OR of 0.94 (95% CI: 0.87-1.01).

**Figure 2.**
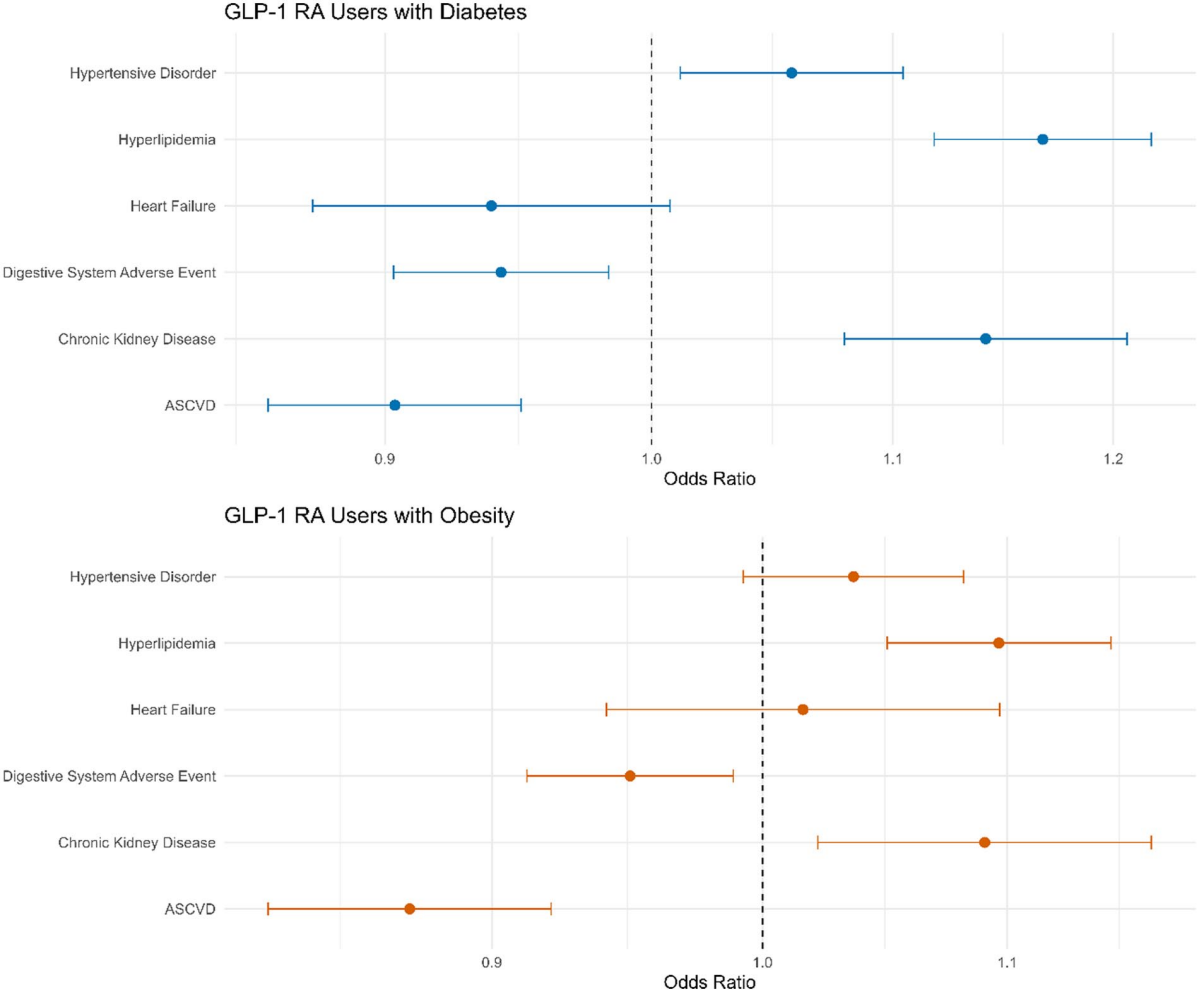
Adjusted ORs for adherence to GLP-1 RAs by comorbidity status and digestive system adverse event status. Forest plot visualizing the adjusted ORs and 95% CIs for adherence associated with individual baseline comorbidities and digestive system adverse event status, corresponding to the data in Table 2.

Among users with obesity, hyperlipidemia (OR: 1.10, 95% CI: 1.05-1.14), and CKD (OR: 1.09, 95% CI: 1.02-1.15), respectively were also positively associated with adherent use in the first year. The ORs for ASCVD and digestive system adverse events were 0.87 (95% CI: 0.83-0.92) and 0.95 (95% CI: 0.91-0.99), respectively. The associations for hypertensive disorder and heart failure were non-statistically significant with estimated OR of 1.04 (95% CI: 0.99-1.08) and 1.02 (95% CI: 0.94-1.10), respectively (see Figure 2).

**Table 2.** Adjusted Odds Ratios for First-Year Adherence to GLP-1 RAs by Baseline Comorbidities or Digestive System Adverse Events

	GLP-1 RA users with diabetes diagnosis			GLP-1 RA users with obesity diagnosis		
Exposure	OR	CI low	CI high	OR	CI low	CI high
Hypertensive disorder	1.06	1.01	1.10	1.04	0.99	1.08
Hyperlipidemia	1.17	1.12	1.22	1.10	1.05	1.15
ASCVD	0.90	0.86	0.95	0.87	0.83	0.92
Heart failure	0.94	0.87	1.01	1.02	0.94	1.10
Chronic kidney disease	1.14	1.08	1.21	1.09	1.02	1.16
Digestive system adverse event	0.94	0.90	0.98	0.95	0.91	0.99

### Brand-Specific Subgroup Analyses

**Figure 3.**
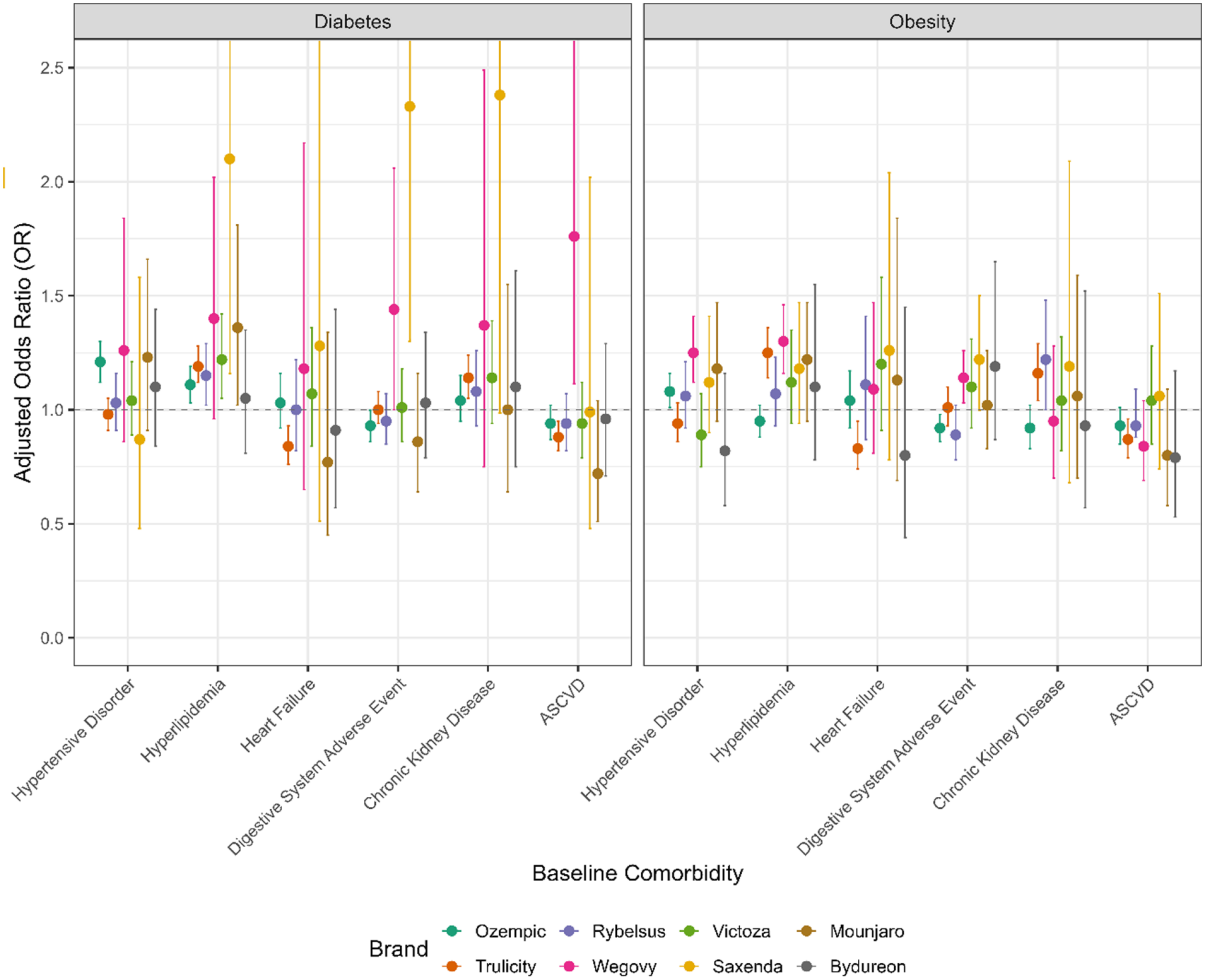
Brand-specific associations between baseline comorbidities and adherence to GLP-1 RAs. This figure displays adjusted ORs and 95% CIs from separate logistic regression models conducted for each GLP-1 RA brand. The results illustrate variation in adherence associations across different medications, analyzed as parallel cohort. Upper-bound of confidence interval exceeding 2.5 shows as 2.5 in the plot above. A complete table including all numbered values on the plot above is available in Appendix Table A1a and A1b.

Brand-specific subgroup analyses across individual GLP-1 RA products generally aligned with the overall trends observed in the primary analysis. However, several product-specific differences were notable. Trulicity was the only drug for which heart failure was statistically significantly associated (OR: 0.84, 95% CI: 0.76-0.93) with lower adherence in the diabetes group. Ozempic demonstrated a non-statistically significant reduction in adherence with baseline digestive system adverse events (OR: 0.93, 95% CI: 0.86-1.0) among the diabetes group. While the reduction in adherence was statistically significant among those with obesity with OR of 0.92 (95% CI: 0.86-0.98) (Figure 3).

## Discussion

This study identified several baseline comorbidities that were consistently associated with improved first-year adherence to GLP-1 RAs among adults with diabetes and/or obesity. Across the overall cohort, the presence of hyperlipidemia and CKD was significantly associated with higher odds of adherence. These associations were observed in both the diabetes and obesity subgroups, suggesting that specific comorbid conditions may influence treatment persistence across different patient populations. Aligning with former studies, patients with diabetes had higher overall adherence than those with obesity, although similar patterns in comorbidity-related associations were observed across both groups.^13,14^

The positive associations between baseline diagnoses of hyperlipidemia and CKD with increased adherence to GLP-1 RAs may reflect patients’ and providers’ recognition of the broader cardiometabolic benefits of these agents. GLP-1 RAs have demonstrated favorable effects on blood pressure, lipid profiles, and renal outcomes in multiple clinical trials and real-world studies, leading to expanded use in patients with overlapping metabolic conditions. As such, individuals with these comorbidities may be more likely to persist with therapy due to perceived or actual improvements in related health domains.^8,9,12^ Moreover, clinicians may be more proactive in initiating and supporting adherence to GLP-1 RAs for patients with complex cardiometabolic profiles, aligning with evolving treatment guidelines that recommend these agents for patients with high cardiovascular and renal risk.^23^ These patterns suggest that anticipated therapeutic benefits may reinforce early treatment persistence among patients with multiple chronic conditions.

In contrast to the positive associations observed for other cardiometabolic conditions, ASCVD demonstrated a consistent negative association with first-year GLP-1 RA adherence across both diabetes and obesity groups. Possible underutilization of cardiologic specialists in GLP-1 RA prescribing may contribute to the findings. Vaduganathan et al^17^ found that less than 5% of GLP-1 RA prescriptions were written by cardiologists, who were significantly less likely to prescribe these agents compared with other specialists. This limited involvement may reflect hesitancy to initiate or continue GLP-1 RA therapy in patients with advanced cardiovascular disease, possibly due to concerns about overlapping treatments, polypharmacy, perceived tolerability issues, or a preference for therapies with more established efficacy in heart failure populations.^24,25^ While similar considerations may also apply to hypertensive disorder and hyperlipidemia, the influence is likely less pronounced, as these conditions are commonly managed in primary care setting. Furthermore, contributory factors such as frailty, disease severity, or competing health priorities, may contribute to lower persistence among these subgroups. Given that GLP-1 RAs have demonstrated cardiovascular benefits in clinical trials, these findings raise important questions about how real-world prescribing and discontinuation decisions are made for patients with existing ASCVD, and whether adherence gaps in this high-risk populations may reflect modifiable clinical practices.^23,24,26^ In addition, digestive system adverse events were found to generally have a negative association with adherence. This may suggest that patients already experiencing gastrointestinal issues commonly associated with GLP-1 RAs (nausea, vomiting, diarrhea, constipation, abdominal pain, or bloating) may have increased difficulty tolerating the potentially multiplied effects experienced as the side effects of new use of the GLP-1 RAs.

When examining results from both the full-sample and brand-specific models, we observed general consistency in directionality, yet important nuances emerged. Several overall associations appear to be driven by the combination of varying, brand-specific relationships that may not reach the threshold to show statistical significance in each group. For example, while ASCVD was associated with reduced adherence among patients with diabetes in the main model, this may reflect a cumulative trend across brands rather than a uniform effect within each specific drug. All brands displayed a non-significant association for reduced adherence with 95% confidence intervals across the null value, except Trulicity (OR: 0.88, 95% CI: 0.82-0.95) and Wegovy (OR: 1.76, 95% CI: 1.10-2.81). Such variation may reflect differences in tolerability, prescribing preferences, or population characteristics across drugs and highlights the importance of examining product-specific patterns when tailoring treatment. This association may be clinically relevant when selecting GLP-1 RAs for patients with underlying GI conditions, especially as drug-specific effects may influence persistence.

Conversely, certain exposure-outcome associations were more pronounced in particular products. Notably, adherence to Trulicity among users with diabetes showed a unique positive association with CKD (OR: 1.14, 95% CI: 1.05-1.24 among patients with diabetes and OR: 1.16, 95% CI: 1.04-1.29 among patients with obesity), suggesting a brand-specific sensitivity to renal comorbidity not observed with other agents. This may be clinically meaningful for prescribers treating patients with CKD, who often face more limited therapeutic options and higher risks of treatment discontinuation. Similarly, Ozempic demonstrated a more pronounced association pattern between adherence and common cardiometabolic comorbidities, particularly among obesity-diagnosed users, possibly reflecting its perceived efficacy in weight management or different patterns of off-label use.^5,6^ This points to potential differences in how this drug is prescribed or tolerated relative to semaglutide’s other formulations, such as Wegovy or Rybelsus.

Although not the primary focus of this analysis, patterns related to Ozempic warrant further attention. As the most frequently prescribed GLP-1 RA in our cohort, Ozempic displayed an adherence pattern in the diabetic group that diverged from the broader diabetes group’s results but aligned more closely with that of obese users of GLP-1 RAs. This pattern could suggest that even among patients carrying a diagnosis of type 1 or type 2 diabetes Ozempic may often be used with weight management as a primary therapeutic goal, irrespective of formal obesity diagnosis. The observed trend aligns with findings from other studies reporting off-label use of Ozempic for weight loss, driven by increased prescriber flexibility, patient demand, and heightened public awareness of weight-reducing effects linked to Ozempic and its generic active ingredient semaglutide in general.^27^ This trend underscores the evolving use of GLP-1 RAs in clinical practice and highlights the need to align treatment goals, such as glycemic control versus weight loss, with individual patient priorities and diagnoses. Moreover, differences in administration route and dosing frequency may influence adherence behavior across GLP-1 RA products. For example, Victoza and Saxenda are administered as a daily subcutaneous injection while Ozempic and Wegovy are administered once a week. In general, we observed no major differences in adherence between these different brands. However, dosage frequency was not specifically adjusted for in our analyses and future studies are necessary to quantify this relationship.

Taken together, these findings emphasize that comorbidities at the time of initiation are not merely background variables but may meaningfully shape early adherence to GLP-1 RAs. They also suggest that different GLP-1 RA products may interact with patient characteristics in distinct ways, which could inform future personalized prescribing practices. Future work should aim to better characterize the clinical mechanisms underlying these associations and determine whether these insights can be used to enhance guideline-based prescribing, improve persistence, and maximize therapeutic benefit. Moreover, these are all realms where clinical pharmacists are increasingly playing a pivotal role.^28^ Understanding these pathways would be essential to supporting sustained medication use and optimizing the therapeutic benefits of GLP-1 RAs in diverse populations.

The strengths of our study include its large, ethnically diverse population-based cohort in UCHDW and its design that intends to capture the timeliness that is most reflective of the current and near-future trend of GLP-1 RAs. This present study had several limitations. First, this study used de-identified outpatient pharmacy dispensing data from the University of California Health system, which has geographic and institutional limitations that may restrict generalizability to broader populations. Second, while the study aimed to capture contemporary GLP-1 RAs use, newer therapies such as Zepbound, approved in late 2023, were not sufficiently represented in the data. Third, some degree of incomplete capture by the data source cannot be ruled out, given adherence was measured using pharmacy dispensing records, which may potentially underestimate adherence for patients who received GLP-1 RAs through non-pharmacy sources such as inpatient settings or clinic samples. Moreover, patients may also get medication from sources that are outside of the traditional health system and such information may not be captured completely in the database. Fourth, the PDC, a standard and widely accepted adherence metric, was calculated based on labeled dosing instructions but does not confirm actual medication use, as patients may refill prescriptions without consistent ingestion.^29^ Finally, as with all observational studies, residual confounding cannot be ruled out, and unmeasured factors such as psychological and behavioral conditions, provider prescribing patterns, patient motivation, and socioeconomic status may have influenced both comorbidity profiles and adherence outcomes.

## Conclusion and Relevance

In this real-world study of GLP-1 RA users, baseline comorbidities were found to influence first-year adherence, with variation across drug types and clinical indications. Conditions such as CKD and hyperlipidemia were associated with higher adherence. Reduced adherence was observed for patients with ASCVD and those that experienced digestive system adverse events. This suggests that certain patient subgroups may experience improved or deteriorated tolerability as well as clinical management.

These findings underscore the need to consider patient characteristics and specific GLP-1 RA products when evaluating adherence. The observed variation by comorbidity and drug subtype highlights the complexity of real-world treatment patterns and the potential for personalized prescribing. Future research should explore mechanisms behind these relationships and assess whether such adherence patterns translate into long-term clinical outcomes.

## Appendix A

**Table A1.** Adjusted Odds Ratios for the Association Between Baseline Comorbidities and 12-Month Adherence to GLP-1 RAs, by Medication Brand.

a. Results Among GLP-1 RA Users With Diabetes								
Condition	Ozempic	Trulicity	Rybelsus	Wegovy	Victoza	Saxenda	Mounjaro	Bydureon
Hypertensive disorder	1.21 [1.12-1.30]	0.98 [0.91-1.05]	1.03 [0.91-1.16]	1.26 [0.86-1.84]	1.04 [0.89-1.21]	0.87 [0.48-1.58]	1.23 [0.91-1.66]	1.10 [0.84-1.44]
Hyperlipidemia	1.11 [1.03-1.19]	1.19 [1.12-1.28]	1.15 [1.02-1.29]	1.40 [0.96-2.02]	1.22 [1.05-1.42]	2.10 [1.17-3.78]	1.36 [1.02-1.81]	1.05 [0.81-1.35]
ASCVD	0.94 [0.87-1.02]	0.88 [0.82-0.95]	0.94 [0.82-1.07]	1.76 [1.10-2.81]	0.94 [0.79-1.12]	0.99 [0.48-2.02]	0.72 [0.51-1.04]	0.96 [0.71-1.29]
Heart failure	1.03 [0.92-1.16]	0.84 [0.76-0.93]	1.00 [0.82-1.22]	1.18 [0.65-2.17]	1.07 [0.84-1.36]	1.28 [0.51-3.18]	0.77 [0.45-1.34]	0.91 [0.57-1.44]
Chronic kidney disease	1.04 [0.95-1.15]	1.14 [1.05-1.24]	1.08 [0.93-1.26]	1.37 [0.75-2.49]	1.14 [0.94-1.39]	2.38 [0.98-5.73]	1.00 [0.64-1.55]	1.10 [0.75-1.61]
Digestive system adverse event	0.93 [0.86-1.00]	1.00 [0.94-1.08]	0.95 [0.85-1.07]	1.44 [1.00-2.06]	1.01 [0.86-1.18]	2.33 [1.29-4.22]	0.86 [0.64-1.16]	1.03 [0.79-1.34]
b. Results Among GLP-1 RA Users With Obesity								
Condition	Ozempic	Trulicity	Rybelsus	Wegovy	Victoza	Saxenda	Mounjaro	Bydureon
Hypertensive disorder	1.08 [1.01-1.16]	0.94 [0.86-1.03]	1.06 [0.92-1.21]	1.25 [1.12-1.41]	0.89 [0.75-1.07]	1.12 [0.90-1.41]	1.18 [0.95-1.47]	0.82 [0.58-1.16]
Hyperlipidemia	0.95 [0.88-1.02]	1.25 [1.14-1.36]	1.07 [0.93-1.23]	1.30 [1.16-1.46]	1.12 [0.94-1.35]	1.18 [0.94-1.47]	1.22 [0.95-1.47]	1.10 [0.78-1.55]
ASCVD	0.93 [0.85-1.01]	0.87 [0.79-0.96]	0.93 [0.88-1.09]	0.84 [0.69-1.04]	1.04 [0.85-1.28]	1.06 [0.74-1.51]	0.80 [0.58-1.09]	0.79 [0.53-1.17]
Heart failure	1.04 [0.92-1.17]	0.83 [0.74-0.95]	1.11 [0.87-1.41]	1.09 [0.81-1.47]	1.20 [0.91-1.58]	1.26 [0.78-2.04]	1.13 [0.69-1.84]	0.80 [0.44-1.45]
Chronic kidney disease	0.92 [0.83-1.02]	1.16 [1.04-1.29]	1.22 [1.00-1.48]	0.95 [0.70-1.28]	1.04 [0.82-1.32]	1.19 [0.68-2.09]	1.06 [0.70-1.59]	0.93 [0.57-1.52]
Digestive system adverse event	0.92 [0.86-0.98]	1.01 [0.93-1.10]	0.89 [0.78-1.02]	1.14 [1.03-1.26]	1.10 [0.92-1.31]	1.22 [1.00-1.50]	1.02 [0.83-1.26]	1.19 [0.87-1.65]

## Appendix B

**Table:** Standardized Diagnostic Codes for Exposures Definitions.

Exposure variable	Value
Hypertensive disorder	Hypertensive disorder (316866), Hypertensive heart disease (442604), Essential hypertension (320128), Renovascular hypertension (317895), Chronic kidney disease due to hypertension (44782429), Hypertensive heart and chronic kidney disease (44784621), Hypertensive heart and renal disease with both (congestive) heart failure and renal failure (439694), Hypertension resistant to drug therapy (44809548), Hypertensive crisis (45768449), Hypertensive urgency (40481896), Hypertensive heart failure (444101), Secondary hypertension (319826), Hypertension secondary to endocrine disorder (4110948), Hypertensive emergency (43020424), Hypertensive heart disease without congestive heart failure (319034), Renal hypertension (443771)
Hyperlipidemia	Hyperlipidemia (432867)
Atherosclerotic cardiovascular disease (ASCVD)	Myocardial infarction (4329847), Angina pectoris (321318), Transient cerebral ischemia (373503), Peripheral arterial disease (3654996), Coronary atherosclerosis (40481919), Atherosclerosis of artery (40479625), Cerebral infarction (443454), Ischemic heart disease (4185932)
Heart failure	Congestive heart failure (319835), Heart failure (316139), Hypertensive heart failure (444101), Chronic diastolic heart failure (40479576), Chronic systolic heart failure (40479192), Chronic congestive heart failure (4229440), Hypertensive heart and renal disease with (congestive) heart failure (439696), Systolic heart failure (443580), Diastolic heart failure (443587)
Chronic kidney disease (CKD)	Chronic kidney disease (46271022), Chronic kidney disease stage 1 (443614), Chronic kidney disease stage 2 (443601), Chronic kidney disease stage 3 (443597), Chronic kidney disease stage 3A (45763854), Chronic kidney disease stage 3B (45763855), Chronic kidney disease stage 4 (443612), Chronic kidney disease stage 5 (443611), Acute kidney injury (197320), Renal failure syndrome (192359), End-stage renal disease (193782), Acute renal failure due to tubular necrosis (45757466), Acute renal failure due to acute cortical necrosis (197329), Acute renal papillary necrosis with renal failure(432961)
Digestive system adverse events	Nausea (31967), Vomiting (441408), Constipation (75860), Diarrhea (196523), Abdominal pain (200219), Abdominal distension, gaseous (4152351)

The exposure definitions in this study were based on standardized diagnosis SNOMED codes. Equivalent and hierarchical relationships between codes were established using the standardized clinical terminology mappings available in the OMOP Common Data Model. Unless otherwise specified, all listed codes and their descendant concepts were included in the definitions.
